# Impact of the COVID-19 Lockdown on Patients with Chronic Tinnitus—Preliminary Results

**DOI:** 10.3390/audiolres12030034

**Published:** 2022-06-15

**Authors:** Alessandra Fioretti, Eleonora Natalini, Gianluigi Triggianese, Rebecca Eibenstein, Anna Maria Angelone, Maria Lauriello, Alberto Eibenstein

**Affiliations:** 1 Tinnitus Center, European Hospital, 00149 Rome, Italy; eleonora.natalini@gmail.com (E.N.); rebecca.eibenstein95@gmail.com (R.E.); alberto.eibenstein@univaq.it (A.E.); 2 Department of Applied Clinical and Biotechnological Sciences, University of Aquila, 67100 L’Aquila, Italy; gianluigitriggianese@gmail.com (G.T.); annamaria.angelone@univaq.it (A.M.A.); maria.lauriello@univaq.it (M.L.)

**Keywords:** tinnitus, COVID-19, anxiety, depression, lockdown, stress, pandemic

## Abstract

The COVID-19 pandemic and the lockdown measures are both causes of psychological distress. The aim of the current study was to evaluate the psychological effects of lockdown measures on patients with subjective chronic tinnitus diagnosed before the COVID-19 pandemic. A sample of *n* = 77 patients with chronic tinnitus was contacted by mail/phone for a survey between June 2021 and September 2021. All patients filled out questionnaires on tinnitus distress (Tinnitus Handicap Inventory, THI), anxiety (Beck Anxiety Inventory, BAI) and depression (Beck Depression Inventory, BDI) and eight items of the Tinnitus Sample Case History (TSCH) about tinnitus history (i.e., loudness, pitch, perception, tinnitus location), stress, and related conditions (noise annoyance, vertigo/dizziness, headache). Forty patients with chronic tinnitus filled out the survey. No significant differences of total THI mean scores (*p* > 0.05) were found compared to the results obtained before the COVID-19 pandemic and after lockdown. Regarding depression and anxiety, the female population showed a significant increase in scores obtained from the BDI (*p* < 0.0170) and the BAI (*p* < 0.049). Only two patients (0.5%) were infected by COVID-19 (positive RT-PCR), and they did not report any worsening of tinnitus. According to the data of the literature, our patients experienced a heterogeneous course of tinnitus, and the severity of tinnitus was not significantly affected by lifestyle changes during the COVID-19 pandemic and lockdown.

## 1. Introduction

Tinnitus is defined as the perception of a sound or sounds in the ear or head without an external source [1]. The prevalence of subjective chronic tinnitus is 10–15% [2,3] of the adult population and tinnitus is very debilitating in 1–2%. Tinnitus is a multifactorial disorder, but in 40% of patients is considered idiopathic [4]. Anxiety and depression are frequently widespread in patients with tinnitus [5,6,7,8,9], but a clear relationship is not still demonstrated.

As shown in the literature, the generation, persistence and recurrence of tinnitus are still debated. Cortical reorganization, secondary to sensory deprivation due to cochlear dysfunction, has been proposed as one of the most frequent causes of tinnitus [2,10]. The avoidance of silence and acoustic masking have been proposed as effective measures to overcome sensory deprivation and to increase symptom masking. Neurological manifestations, such as olfactory and taste alterations, headaches, hearing loss, tinnitus and dizziness, have been reported in COVID-19 patients [11,12]. Viola et al. [13] reported a 23.2% prevalence of subjective tinnitus in a sample of 185 COVID-19 patients using an online questionnaire. A systematic review by Beukes et al. [14] suggested that the pooled estimated prevalence of tinnitus post-COVID-19 is 8% (CI: 5 to 13%). The management of the COVID-19 pandemic led the Italian government to declare, on 9 March 2020, the prohibition of any movement within national territory, except for work/health reasons, and the obligation to stay at home as much as possible. Subsequently, the lockdown measures were relaxed in alternating phases with different regional trends. It is reasonable to think that, during the lockdown, the absence of environmental masking sounds from everyday life may have increased the perception of tinnitus. Additionally, the stress experienced during the pandemic could be included as an additional potential risk factor for worsening tinnitus.

Mazza et al. [15] found a significant increase in psychological distress in the general Italian population during the COVID-19 pandemic. Specifically, individuals with previous situations of high stress reported higher levels of anxiety and depression; the same data emerged for people with chronic diseases, probably because COVID-19 has increased a greater sense of vulnerability and fear. Casagrande et al. [16] focused on the quality of sleep during the COVID-19 emergency, reporting an increase in sleep disorders and consequent psychological problems, particularly the risk of the onset of symptoms linked to post-traumatic stress disorder (PTSD).

The emotional impact of the pandemic has also led to major changes in daily life activities, such as physical exercise, sexuality, and nutrition [17]. The results of the studies carried out on the Italian population during COVID-19 pandemic are in line with the data collected in other countries [18,19,20,21], and they highlight the need for greater attention to mental health.

The aim of this work was to investigate, through a survey, the psychological effects of lockdown measures during the COVID-19 pandemic on patients with subjective chronic tinnitus diagnosed before the pandemic.

## 2. Materials and Methods

### 2.1. Sample and Setting

A sample of 77 patients with chronic tinnitus evaluated before the COVID-19 pandemic at the Tinnitus Center of the European Hospital in Rome was selected. All the patients were contacted by mail/phone for a survey between June 2021 and September 2021. The survey included validated questionnaires and some items of the Tinnitus Sample Case History (TSCH). The patients were evaluated by an ENT specialist and a psychologist, and they filled out the questionnaires before the COVID-19 pandemic. Inclusion criteria were: (a) chronic tinnitus (more than 6 months); (b) age over 18 years; (c) speaking Italian fluently; and (d) no apparent cognitive impairment or major psychiatric/neurological disorder (schizophrenia, Alzheimer’s disease, Parkinson’s disease). All procedures performed in this study involving human participants were in accordance with the ethical standards of the institutional and/or national research committee and with the 1964 Helsinki declaration and its later amendments or comparable ethical standards. The study was approved by the Internal Review Board of the University of L’Aquila (protocol code 89132 of 26 July 2021). Participation was on a voluntary basis and every patient gave written consent to the use of anonymity data provided in the responses to the questionnaires. The treatment of the patients before March 2020 was based on personalized therapies and included cognitive behavioral therapy (individual sessions), hearing aids, otorhinolaryngological follow up, pharmacotherapy, and physiotherapeutic sessions.

### 2.2. Measures

All patients filled out the following standardized questionnaires: TSCH, the Tinnitus Handicap Inventory (THI); the Beck Anxiety Inventory (BAI); and the Beck Depression Inventory (BDI). Responses of the questionnaires were recorded in an Excel spreadsheet. Access to the response spreadsheet was limited to the principal investigators. Two adjunctive questions were focused on COVID-19: (1) Have you tested positive for COVID-19? yes/no; (2) have lifestyle changes associated with containing COVID-19 affected your tinnitus? yes, it is worse/yes, the tinnitus got worse for a short time/no.

#### 2.2.1. Standardized Questionnaires

**Tinnitus Sample Case History (TSCH)**

The TSCH is a standardized questionnaire of 35 items developed by the Tinnitus Research Initiative to collect sociodemographic and clinical data in tinnitus research [22]. The 8 items of the Italian version of the TSCH used in this survey were about tinnitus history (i.e., loudness, pitch, perception, tinnitus location), stress, and related conditions (noise annoyance, vertigo/dizziness, headache).

**Tinnitus Handicap Inventory (THI)**

The THI [23] is a self-report questionnaire widely used in tinnitus research to assess the impact of tinnitus in daily life. It consists of 25 items and 3 subscales (functional, emotional, and catastrophic). Based on the total score of the Italian THI version [24], tinnitus severity can be graded from slight (grade 1) to catastrophic (grade 5). A THI score > 36 was considered to indicate decompensated tinnitus [25,26].

**Beck Anxiety Inventory (BAI)**

The BAI [27] is a self-administered tool of 21 items used to assess the severity of anxiety over the last seven days. Every item can be rated on a 4-point scale with a score ranging from 0 (‘not at all’) to 3 (‘severely’). Based on the total score, anxiety can be classified as minimal (0–7), mild (8–15), moderate (16–25), and severe (26–63).

**Beck Depression Inventory (BDI)**

The BDI-II [28] is a self-report instrument of 21 items to assess depressive symptoms over the previous two weeks. Based on the total score, depression can be classified as minimal (0–13), mild (14–19), moderate (20–28), or severe (29–63).

#### 2.2.2. Tonal Audiometry

All patients were tested with pure tonal audiometry during the first evaluation (2018–2019). Normal hearing was defined by threshold < 25 dB HL in all frequencies tested between 250 and 8000 Hz.

### 2.3. Statistical Analyses

A descriptive analysis was performed by calculating the relative frequencies for categorical variables: tinnitus perception, tinnitus location, tinnitus loudness varying from day to day, stress effect, tinnitus pitch, hyperacusis, headache, and vertigo. The comparison between these variables before COVID-19 and after the lockdown period was effectuated with the McNemar test.

THI, BDI and BAI total scores were considered as continuous variables and are presented as means and standard deviations. The mean score of these questionnaires before COVID-19 and after the lockdown period was evaluated with a t-test for paired data. The Fischer exact test was used to examine the possible differences between different grades for the THI, BDI and BAI before COVID-19 and after the lockdown.

The t-test for paired data was used to analyze the difference between sex and age under 50 and over 51 years before COVID-19 and after the lockdown period. Finally, the differences of THI, BDI and BAI scores before COVID-19 and after the lockdown periods were also tested in males, females, and patients aged under and over 50 using the t-test for paired data.

A *p*-value of less than 0.05 was considered significant. The data were analyzed using the Stata 15 software (Stata Corp LP, College Station, TX, USA).

## 3. Results

Thirty-seven patients did not answer the phone/mail request or did not have time to answer the survey. A total of *n* = 40 patients (52.5% male) participated in the survey and were included in the analyses. The mean age was 47.8 (SD: 13.2) years. Only two patients (0.5%) were infected with COVID-19 (positive RT-PCR), and they did not report any worsening of tinnitus. Lifestyle changes associated with containing COVID-19 did not affect tinnitus in 33 patients (82.5%). However, 80% of patients described their tinnitus as continuously present after lockdown (*p* < 0.00019), and 77.5% of patients described their tinnitus as bilateral or central after lockdown (*p* < 0.0041). Details on patient sociodemographic characteristics before COVID-19 and after lockdown can be found in Table 1. We found that 65% of patients had increased tinnitus related to stress before COVID-19 and 77.5% of patients had increased tinnitus related to stress after lockdown (*p* < 0.0005) (Table 1).

**Figure 1:**
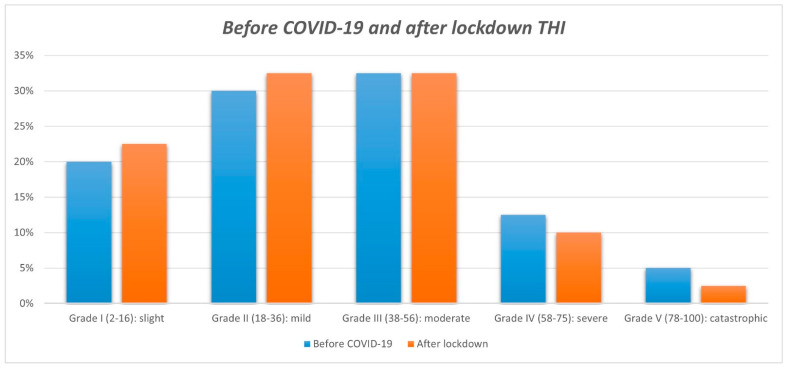
THI grades before COVID-19 and after lockdown.

Based on the pure tone audiometry (250-8.000 Hz) before COVID-19, 26 patients had hearing loss. According to the total THI score, 22.5% of the patients were classified as very mild (grade I), 32.5% as mild (grade II), 32.5% as moderate (grade III), 10% as severe (grade IV), and 2.5% as very severe (grade V) after lockdown (Figure 1). Moreover, 47.5% of patients reported decompensated tinnitus after lockdown (THI > 36). Based on the data obtained before COVID-19 and post-lockdown, no significant differences were found in the total THI mean scores

### 3.1. Association of Tinnitus Distress with Depression and Anxiety

#### 3.1.1. Depression

Regarding depression, based on the BDI score, 24 patients (60%) showed no symptoms or minimal symptoms, while 11 patients (27.5%) showed mild symptoms, 4 patients (10%) showed moderate depressive symptoms, and 1 patient (2.5%) showed severe symptoms after lockdown. According to the total BDI mean scores before COVID-19 and after lockdown, no significant differences were found.

#### 3.1.2. Anxiety

Regarding anxiety, based on the BAI scores, 47.5% of patients showed no symptoms or mild symptoms, 22.5% of patients showed mild symptoms, 25.5% of patients showed moderate symptoms, and 5% showed severe symptoms after lockdown. According to the data obtained before COVID-19 and after lockdown, no significant differences were found in the total BAI mean scores.

The mean scores and standard deviation of the THI, BAI and BDI before COVID-19 and after lockdown were analyzed based on gender and age. The direct comparison of the THI scores before and after lockdown revealed a decrease in THI scores in males (*p* < 0.0481). Regarding depression and anxiety, the female population showed a significant increase in scores obtained from the BDI (*p* < 0.0170) and the BAI (*p* < 0.049).

## 4. Discussion

In this study, we investigated the effect of lockdown measures during the COVID-19 pandemic on 40 tinnitus patients previously assessed in our clinic. In addition to tinnitus severity and handicap level evaluated with TSCH and the THI, depression and anxiety were assessed with the BDI and the BAI. We are aware that, during the lockdown period from March to May 2020, it was impossible to carry out almost all activities, including those related to the external environment and work activities. Our survey was performed one year after the beginning of the lockdown and the answers might be conditioned by the improvement of the pandemic restrictions between June and September 2021. After the lockdown period, patients with continuous tinnitus after previously having intermittent tinnitus increased from 67.5% to 80% (*p* < 0.00019).

### 4.1. Tinnitus Distress and Lifestyle Changes Related to the COVID-19 Pandemic

The increase in tinnitus distress during the pandemic has been reported by other authors [29,30]. In our study, a total of 77.5% of patients experienced worsening tinnitus related to stress, but 82.5% did not report any relationship between lifestyle changes and tinnitus.

The use of THI allowed us to evaluate how the impact of tinnitus on quality of life changed among patients. High standard deviation values were recorded, showing that many patients reported an increase in tinnitus distress, although others reported a decrease in tinnitus distress. Beukes et al. [31], in a cross-sectional study during the COVID-19 pandemic on 3.103 patients with pre-existing tinnitus, found that tinnitus was more bothersome for 32% of respondents, particularly for females and younger adults, and found no change in tinnitus for 67% of respondents. The authors also suggest the contribution of external factors such as financial worries, social isolation, and reduced levels of exercise to tinnitus being more bothersome during the COVID-19 pandemic. In the present study, based on the THI scores, no significant evidence was found distinguishing data according to age. Male data reported a decrease in mean THI scores before COVID-19 and after lockdown (*p* < 0.041), showing that these patients did not have a worse quality of life related to tinnitus during the lockdown.

### 4.2. No Statistically Significant Change in Depressive Symptoms

Significantly, 60% of tinnitus patients showed minimal grades of depression based on mean BDI score after lockdown. Comparing data before the COVID-19 pandemic and after the lockdown periods, there was a reduction in patients experiencing minimal depressive symptoms (from 72.5% to 60%). In addition, there was an increase in the number of patients who began to experience the psychological impact of tinnitus, i.e., depressive symptoms. This is supported by an increase in patients with mild depression, from 15% to 27.5% after lockdown. Based on the present data it is not clear if the low mood was due to tinnitus or to the lifestyle changes after lockdown. Schlee et al. reported no significant changes in depressive symptoms, as measured by the Major Depression Inventory, in tinnitus patients during the pandemic [29].

### 4.3. No Statistically Significant Change in Anxiety

Comparing data before the COVID-19 pandemic and after the lockdown periods, the percentage of patients experiencing minimal anxiety symptoms was unchanged (47.5%): 25.5% of patients reported moderate anxiety after lockdown (20% before the COVID-19 pandemic). A worsening of mean BAI score was reported among patients over 51 years of age, but this was not statistically significant. Li et al. [32] showed that, during the pandemic, negative emotions such as anxiety, depression, indignation, and sensitivity to social risks increased, while the scores of positive emotions and life satisfaction decreased in the general population. Other authors support the role of anxiety as a risk factor of tinnitus during the pandemic [14,31,33], but also as a worsening factor for pre-existing tinnitus. The relationship between anxiety, depression and tinnitus has been evidenced, demonstrating a negative impact on the life quality of patients. Specific interventions on both anxiety and depression in order to avoid the aggravation of pre-existing chronic tinnitus are recommended.

Regarding depression and anxiety, the female population showed a significant increase in scores obtained from the BDI questionnaire (*p* < 0.0170) and the BAI questionnaire (*p* < 0.049), a sign of a strong changes in emotional state induced by the pandemic. The results are in line with those of previous research that have highlighted greater psychological distress for women during the pandemic [15,21,34]. The data, therefore, do not seem to be related to tinnitus, but to a general emotional vulnerability in the female population in the presence of stressful situations, such as the COVID-19 emergency. Anxiety, sleep disorders, and psychiatric features might appear even in the absence of the COVID-19 pandemic, and be related to other chronic diseases such as migraines. As reported by Di Stefano et al., patients with migraines (almost always females) worsening during lockdown presented higher BDI scores and impaired sleep, as demonstrated by Insomnia Severity Index scores during the lockdown [35].

Clinicians should be aware that tinnitus might be more problematic following the COVID-19 pandemic due to the presence of additional factors such as stress and anxiety. Based on the small number of patients infected by COVID-19 in our study, we cannot state if COVID-19 infection influences the outcome of tinnitus. A final consideration must go to patients’ noise exposure levels before the pandemic situation, and the noise exposure experienced during home confinement. The majority of our patients lived in an urban environment during the lockdown.

COVID-19 pandemic had the benefit of reducing noise exposure during the lockdown [36]. Reducing the noise sources outside by cutting down traffic flows depended on a lot of parameters, mainly on where the patients lived. Noise exposure in working environments obviously depends on the working activities. Exposure to noise is associated with sleep disorders, awakenings [37], learning impairments [38,39], hypertension, ischemic heart disease [40,41], diastolic blood pressure [42], a reduction in working performance [43,44], and annoyance [45]. Major sources found to be the most impactful on human lifestyle include road traffic [46,47,48], railway traffic [49], airports [50], and port activities [51]. The relationship between environmental noise and COVID-19 is well documented in the literature, as some subjects are more sensitive to noise in quieter environments [52,53].

High-quality studies with bigger sample sizes are needed to investigate the effects of COVID-19 infection and lockdown measures during the COVID-19 pandemic, as well as to understand long-term effects on patients with chronic tinnitus.

### 4.4. Limits of This Study

This preliminary study presents several limitations that should be considered. The first is the low number of patients included in the study and the consequent decrease in statistical power. The second is the absence of clinical re-evaluation of these patients (otoscopy, audiologic examination). The third is that we mainly report lockdown effect and not COVID-19 effect, due to the limited number of patients who tested positive for COVID-19. The influence on tinnitus of insomnia, mourning, job loss, and individual variables such as lifestyle change after lockdown, stress resilience, and financial worries during the pandemic were not evaluated. So, we do not have sufficient elements to differentiate the natural course of tinnitus and tinnitus changes due to lockdown measures. The absence of noise exposure evaluation also represents a limitation and a bias of this work.

## 5. Conclusions

According to our data, our patients experienced a heterogeneity of individual perceptions of tinnitus related to lifestyle changes during the COVID-19 pandemic and lockdown. These results may be affected by other factors, such as stress, noise exposure, anxiety, and depression. Based on the THI scores, no significant differences were found comparing the results obtained before the COVID-19 pandemic and after lockdown, nor were there any distinguishing data according to age difference. Male patients did not develop a worse quality of life related to tinnitus during the lockdown. Anxiety and depression in tinnitus patients were more prevalent among women.

## Figures and Tables

**Table 1:** Sociodemographic and clinical data before COVID-19 and after lockdown.

	*Before COVID-19*		*After Lockdown*		
	(*n* = 40)		(*n* = 40)		
	*n*	*%*	*n*	*%*	p-value *
Tinnitus perception					
Constant	27	67.5%	32	80%	*0.00019*
Intermittent	11	27.5%	8	20%	
Missing	2	5%	-	-	
Tinnitus location					
Only one ear (right or left)	14	35%	9	22.5%	*0.0041*
Both ears and inside the head	26	65%	31	77.5%	
*Missing*	-	-	-	-	
Tinnitus loudness varying from day to day *Yes*	27	67.5%	29	72,5%	*0.0136*
*No*	13	32.5%	11	27.5%	
*Missing*	-	-	-	-	
Stress effect					
Increased tinnitus	26	65%	31	77.5%	*0.0005*
No effect	9	22.5%	9	22.5%	
*Missing*	5	12.5%	-	-	
Tinnitus pitch					
Very high and high	29	72.5%	24	60%	*0.0090*
Medium and low	9	22.5%	16	40%	
*Missing*	2	5%	-	-	
Intolerance to sound					
Never rarely sometimes	28	70%	29	72.5%	*0.0012*
Often always	9	22.5%	11	27.5%	
*Missing*	3	7.5%	-	-	
Headache *Yes*	19	47.5%	17	42.5%	*ns*
*No*	19	47.5%	23	57.5%	
*Missing*	2	5%	-	-	
Vertigo/dizziness *Yes*	10	25%	13	32.5%	*0.026*
*No*	27	67.5%	27	67.5%	
*Missing*	3	7.5%	-	-	

* McNemar Test; *ns* = not significant.

## Data Availability

The data presented in this study are available on request from the corresponding author AF. The data are not publicly available since the consent of the patients to do so was not obtained.

## References

[1] Cima R.F.F., Mazurek B., Haider H., Kikidis D., Lapira A., Norena A., Hoare D.J. (2019). A multidisciplinary European guideline for tinnitus: Diagnostics, assessment, and treatment. HNO.

[2] Baguley D., McFerran D., Hall D. (2013). Tinnitus. Lancet.

[3] Biswas R., Hall D.A. (2021). Prevalence, Incidence, and Risk Factors for Tinnitus. Curr. Top. Behav. Neurosci..

[4] Henry J.A., Dennis K.C., Schechter M.A. (2005). General review of tinnitus: Prevalence, mechanisms, effects, and management. J. Speech Lang. Hear. Res..

[5] Salazar J.W., Meisel K., Smith E.R., Quiggle A., Mccoy D.B., Amans M.R. (2019). Depression in Patients with Tinnitus: A Systematic Review. Otolaryngol. Head Neck Surg..

[6] Trevis K.J., McLachlan N.M., Wilson S.J. (2018). A systematic review and meta-analysis of psychological functioning in chronic tinnitus. Clin. Psychol. Rev..

[7] Pattyn T., Van Den Eede F., Vanneste S., Cassiers L., Veltman D.J., Van De Heyning P., Sabbe B.C.G. (2016). Tinnitus and anxiety disorders: A review. Hear. Res..

[8] Durai M., Searchfield G. (2016). Anxiety and depression, personality traits relevant to tinnitus: A scoping review. Int. J. Audiol..

[9] Gomaa M.A., Elmagd M.H., Elbadry M.M., Kader R.M. (2014). Depression, Anxiety and Stress Scale in patients with tinnitus and hearing loss. Eur. Arch. Otorhinolaryngol..

[10] Shore S.E., Roberts L.E., Langguth B. (2016). Maladaptive plasticity in tinnitus—Triggers, mechanisms and treatment. Nat. Rev. Neurol..

[11] Munro K.J., Uus K., Almufarrij I., Chaudhuri N., Yioe V. (2020). Persistent self-reported changes in hearing and tinnitus in post-hospitalisation COVID-19 cases. Int. J. Audiol..

[12] Almufarrij I., Munro K.J. (2021). One year on: An updated systematic review of SARS-CoV-2, COVID-19 and audio-vestibular symptoms. Int. J. Audiol..

[13] Viola P., Ralli M., Pisani D., Malanga D., Sculco D., Messina L., Laria C., Aragona T., Leopardi G., Ursini F. (2020). Tinnitus and equilibrium disorders in COVID-19 patients: Preliminary results. Eur. Arch. Otorhinolaryngol..

[14] Beukes E., Ulep A., Eubank T., Manchaiah V. (2021). The Impact of COVID-19 and the Pandemic on Tinnitus: A Systematic Review. J. Clin. Med..

[15] Mazza C., Ricci E., Biondi S., Colasanti M., Ferracuti S., Napoli C., Roma P.A. (2020). Nationwide Survey of Psychological Distress among Italian People during the COVID-19 Pandemic: Immediate Psychological Responses and Associated Factors. Int. J. Environ. Res. Public Health.

[16] Casagrande M., Favieri F., Tambelli R., Forte G. (2020). The enemy who sealed the world: Effects quarantine due to the COVID-19 on sleep quality, anxiety, and psychological distress in the Italian population. Sleep Med..

[17] Ferrucci R., Averna A., Marino D., Reitano M.R., Ruggiero F., Mameli F., Dini M., Poletti B., Barbieri S., Priori A. (2020). Psychological Impact During the First Outbreak of COVID-19 in Italy. Front. Psychiatry.

[18] Huang Y., Zhao N. (2020). Generalized anxiety disorder, depressive symptoms and sleep quality during COVID-19 outbreak in China: A web-based cross-sectional survey. Psychiatry Res..

[19] Ozamiz-Etxebarria N., Dosil-Santamaria M., Picaza-Gorrochategui M., Idoiaga-Mondragon N. (2020). Stress, anxiety, and depression levels in the initial stage of the COVID-19 outbreak in a population sample in the northern Spain. Cad. Saude Publica.

[20] Roy D., Tripathy S., Kar S.K., Sharma N., Verma S.K., Kaushal V. (2020). Study of knowledge, attitude, anxiety & perceived mental healthcare need in Indian population during COVID-19 pandemic. Asian J. Psychiatr..

[21] Wang C., Pan R., Wan X., Tan Y., Xu L., Ho C.S., Ho R.C. (2020). Immediate psychological responses and associated factors during the initial stage of the 2019 coronavirus disease (COVID-19) epidemic among the general population in China. Int. J. Environ. Res. Public Health.

[22] Langguth B., Goodey R., Azevedo A., Bjorne A., Cacace A., Crocetti A., Del Bo L., De Ridder D., Diges I., Elbert T. (2007). Consensus for tinnitus patient assessment and treatment outcome measurement: Tinnitus Research Initiative meeting, Regensburg, July 2006. Prog. Brain Res..

[23] Newman C.W., Jacobson G.P., Spitzer J.B. (1996). Development of the Tinnitus Handicap Inventory. Arch. Otolaryngol. Head Neck Surg..

[24] Passi S., Ralli G., Capparelli E., Mammone A., Scacciatelli D., Cianfrone G. (2008). The THI questionnaire: Psychometric data for reliability and validity of the Italian version. Int. Tinnitus J..

[25] Salviati M., Macri F., Terlizzi S., Melcore C., Provenzano A., Capparelli E., Altissimi G., Cianfrone G. (2013). The Tinnitus Handicap Inventory as a screening test for psychiatric comorbidity in patients with tinnitus. Psychosomatics.

[26] Altissimi G., Salviati M., Turchetta R., Orlando M.P., Greco A., De Vincentiis M., Ciofalo A., Marinelli C., Testugini V., Mazzei F. (2016). When alarm bells ring: Emergency tinnitus. Eur. Rev. Med. Pharmacol. Sci..

[27] Beck A.T., Epstein N., Brown G., Steer R.A. (1988). An inventory for measuring clinical anxiety: Psychometric properties. J. Consult. Clin. Psychol..

[28] Beck A.T., Steer R.A., Brown G.K. (1996). Manual for the Beck Depression Inventory—II.

[29] Schlee W., Hølleland S., Bulla J., Simoes J., Neff P., Schoisswohl S., Woelflick S., Schecklmann M., Schiller A., Staudinger S. (2020). The Effect of Environmental Stressors on Tinnitus: A Prospective Longitudinal Study on the Impact of the COVID-19 Pandemic. J. Clin. Med..

[30] Anzivino R., Sciancalepore P.I., Petrone P., D’Elia A., Petrone D., Quaranta N. (2021). Tinnitus revival during COVID-19 lockdown: How to deal with it?. Eur. Arch. Otorhinolaryngol..

[31] Beukes E.W., Baguley D.M., Jacquemin L., Lourenco M.P.C.G., Allen P.M., Onozuka J., Stockdale D., Kaldo V., Andersson G., Manchaiah V. (2020). Changes in Tinnitus Experiences during the COVID-19 Pandemic. Front. Public Health.

[32] Li S., Wang Y., Xue J., Zhao N. (2020). The impact of COVID-19 epidemic declaration on psychological consequences: A study on active Weibo users. Int. J. Environ. Res. Public Health.

[33] Xia L., He G., Feng Y., Yu X., Zhao X., Yin S., Chen Z., Wang J., Fan J., Dong C. (2021). COVID-19 associated anxiety enhances tinnitus. PLoS ONE.

[34] Qiu J., Shen B., Zhao M., Wang Z., Xie B., Xu Y. (2020). A nationwide survey of psychological distress among Chinese people in the COVID-19 epidemic: Implication and policy recommendations. Gen. Psychiatr..

[35] Di Stefano V., Ornello R., Gagliardo A., Torrente A., Illuminato E., Caponnetto V., Frattale I., Golini R., Di Felice C., Graziano F. (2021). Social Distancing in Chronic Migraine during the COVID-19 Outbreak: Results from a Multicenter Observational Study. Nutrients.

[36] Asensio C., Aumond P., Can A., Gascó L., Lercher P., Wunderli J.-M., Lavandier C., de Arcas G., Ribeiro C., Muñoz P. (2020). A Taxonomy Proposal for the Assessment of the Changes in Soundscape Resulting from the COVID-19 Lockdown. Int. J. Environ. Res. Public Health.

[37] Muzet A. (2007). Environmental noise, sleep and health. Sleep Med. Rev..

[38] Zacarías F.F., Molina R.H., Ancela J.L.C., López S.L., Ojembarrena A.A. (2013). Noise exposure in preterm infants treated with respiratory support using neonatal helmets. Acta Acust. United Acust..

[39] Erickson L.C., Newman R.S. (2017). Influences of background noise on infants and children. Curr. Dir. Psychol. Sci..

[40] Dratva J., Phuleria H.C., Foraster M., Gaspoz J.-M., Keidel D., Künzli N., Liu L.-J.S., Pons M., Zemp E., Gerbase M.W. (2012). Transportation noise and blood pressure in a population-based sample of adults. Environ. Health Perspect..

[41] Babisch W., Beule B., Schust M., Kersten N., Ising H. (2005). Traffic noise and risk of myocardial infarction. Epidemiology.

[42] Petri D., Licitra G., Vigotti M.A., Fredianelli L. (2021). Effects of Exposure to Road, Railway, Airport and Recreational Noise on Blood Pressure and Hypertension. Int. J. Environ. Res. Public Health.

[43] Vukić L., Fredianelli L., Plazibat V. (2021). Seafarers’ Perception and Attitudes towards Noise Emission on Board Ships. Int. J. Environ. Res. Public Health.

[44] Rossi L., Prato A., Lesina L., Schiavi A. (2018). Effects of low-frequency noise on human cognitive performances in laboratory. Build. Acoust..

[45] Miedema H.M.E., Oudshoorn C.G.M. (2001). Annoyance from transportation noise: Relationships with exposure metrics DNL and DENL and their confidence intervals. Environ. Health Perspect..

[46] Cueto J.L., Petrovici A.M., Hernández R., Fernández F. (2017). Analysis of the Impact of Bus Signal Priority on Urban Noise. Acta Acust. United Acust..

[47] Morley D.W., de Hoogh K., Fecht D., Fabbri F., Bell M., Goodman P.S., Elliott P., Hodgson S., Hansell A.L., Gulliver J. (2015). International scale implementation of the CNOSSOS-EU road traffic noise prediction model for epidemiological studies. Environ. Pollut..

[48] Ruiz-Padillo A., Ruiz D.P., Torija A.J., Ramos-Ridao Á. (2016). Selection of suitable alternatives to reduce the environmental impact of road traffic noise using a fuzzy multi-criteria decision model. Environ. Impact Assess. Rev..

[49] Bunn F., Trombetta Zannin P.H. (2016). Assessment of railway noise in an urban setting. Appl. Acoust..

[50] Iglesias-Merchan C., Diaz-Balteiro L., Soliño M. (2015). Transportation planning and quiet natural areas preservation: Aircraft overflights noise assessment in a National Park. Transp. Res. Part D Transp. Environ..

[51] Nastasi M., Fredianelli L., Bernardini M., Teti L., Fidecaro F., Licitra G. (2020). Parameters affecting noise emitted by ships moving in port areas. Sustainability.

[52] Tong H., Aletta F., Mitchell A., Oberman T., Kang J. (2021). Increases in noise complaints during the COVID-19 lockdown in Spring 2020: A case study in Greater London, UK. Sci. Total Environ..

[53] Mishra A., Das S., Singh D., Maurya A.K. (2021). Effect of COVID-19 lockdown on noise pollution levels in an Indian city: A case study of Kanpur. Environ. Sci. Pollut. Res..

